# Adaptation Time as a Determinant of the Dosimetric Effectiveness of Online Adaptive Radiotherapy for Bladder Cancer

**DOI:** 10.3390/cancers15235629

**Published:** 2023-11-29

**Authors:** Aymane Khouya, Christoph Pöttgen, Christian Hoffmann, Toke Printz Ringbaek, Wolfgang Lübcke, Frank Indenkämpen, Maja Guberina, Nika Guberina, Thomas Gauler, Martin Stuschke, Alina Santiago Garcia

**Affiliations:** 1 Department of Radiotherapy, University of Duisburg-Essen, Hufelandstraße 55, 45147 Essen, Germany; 2 German Cancer Consortium (DKTK), Partner Site University Hospital Essen, University of Duisburg-Essen, 45147 Essen, Germany

**Keywords:** online adaptive radiotherapy, bladder cancer, intrafractional motion, bladder deformation

## Abstract

**Simple Summary:**

Online adaptive radiotherapy (oART) can potentially reduce the required planning target volume (PTV) margins for clinical target volume (CTV) coverage. To achieve this advantage, it is crucial that intrafractional CTV deformation during adaptation remains smaller than deformation from fraction to fraction. In this study, we analyzed the dosimetric effects of intrafractional CTV deformations using a time-dependent CTV deformation model based on cone beam CT data generated before and after adaptation. Data were obtained from nine patients undergoing online adaptive definitive focal radiochemotherapy as part of a clinical registry. In half of the treatment series, a significant time dependence in the required margin to maintain an effective uniform dose within the CTV was observed. Extending the adaptation time beyond 10 min by an additional 5 min necessitated a 1.9 ± 0.24 mm increase in the margin. This underscores the importance of minimizing adaptation time as it significantly influences the precision of oART.

**Abstract:**

Interfraction anatomic deformations decrease the precision of radiotherapy, which can be improved by online adaptive radiation therapy (oART). However, oART takes time, allowing intrafractional deformations. In this study on focal radiotherapy for bladder cancer, we analyzed the time effect of oART on the equivalent uniform dose in the CTV (EUD_CTV_) per fraction and for the accumulated dose distribution over a treatment series as measure of effectiveness. A time-dependent digital CTV model was built from deformable image registration (DIR) between pre- and post-adaptation imaging. The model was highly dose fraction-specific. Planning target volume (PTV) margins were varied by shrinking the clinical PTV to obtain the margin-specific CTV. The EUD_CTV_ per fraction decreased by—4.4 ± 0.9% of prescribed dose per min in treatment series with a steeper than average time dependency of EUD_CTV_. The EUD_CTV_ for DIR-based accumulated dose distributions over a treatment series was significantly dependent on adaptation time and PTV margin (*p* < 0.0001, Chi2 test for each variable). Increasing adaptation times larger than 10 min by five minutes requires a 1.9 ± 0.24 mm additional margin to maintain EUD_CTV_ for a treatment series. Adaptation time is an important determinant of the precision of oART for one half of the bladder cancer patients, and it should be aimed at to be minimized.

## 1. Introduction

Online adaptive radiotherapy (oART) has been studied in several clinical indications such as prostate, head and neck, lung and cervix treatments, demonstrating enhanced target coverage and sparing of organs at risk (OAR) [1,2,3,4,5,6,7]. While offline ART involves generating new treatment plans for subsequent fractions, oART takes a step further by enabling plan adjustments in real time while the patient is on the treatment couch. This approach ensures that the treatment plan is tailored to the patient’s anatomy captured by pre-adaptation imaging. Currently, oART is available with cone beam computed tomography (CBCT)- or magnetic resonance imaging (MRI)-equipped linear accelerators (LINACs). These techniques have demonstrated variations in treatment duration. Using an MRI-based LINAC in adapt-to-shape (ATS) mode [8], treatment durations ranging from 19.1 to 106.4 min have been reported [9,10,11], while with a CBCT-based LINAC, durations of 11.9 to 34.1 min have been reported [12,13]. During this adaptation time, profound intrafractional deformations occur, influencing the delivered dose to the CTV due to variations in bladder-shape-dependent CTV deformations. Bladder volume increases during radiotherapy on average by 2.1–4.0 mL/min [14,15,16]. Bladder wall displacements from treatment fraction to treatment fraction have been measured, indicating the need for PTV margins of 15–25 mm for bladder cancer treatment without online adaptation [17,18,19]. While for whole-bladder treatments, patients are usually treated with an empty bladder, focal bladder cancer treatments can benefit from a filled bladder, which allows for better sparing of non-involved bladder walls [20,21,22]. Four studies on oART have also been reported on bladder cancer. In two studies, the whole bladder was included into the target volume, and patients were treated with an empty bladder [23,24]; in the other two studies, focal radiotherapy to the bladder tumor was delivered using simultaneous integrated or sequential boost [25,26]. The latter studies used PTV margins for the boost CTV between 5 and 10 mm.

The aim of this study is to investigate the impact of adaptation time between the initial imaging study, as a basis for oART on the delivered dose to the CTV, derived from imaging immediately after adaptation and just before treatment execution for focal radiotherapy of bladder cancer with a filled bladder. The adaptation time dependence of the effective uniform dose in the CTV (EUDCTV) was studied from fraction to fraction interfractional per treatment phase or series using a fixed isotropic PTV margin of 5 mm instead of individualized clinical margins. In addition, a digital CTV and bladder phantom was built to capture the fraction-specific deformations of the bladder or CTV between both imaging studies, assuming linear volume growth over time. Employing deformable image-based dose accumulation, this study examines the influence of adaptation time and the PTV margin around the CTV, which was varied from 2 mm to 14 mm, on the EUDCTV for the entire treatment series.

## 2. Materials and Methods

### 2.1. Online Adaptive Radiotherapy

Consecutive patients with bladder carcinoma treated prior to March 2023 with oART treatment using the Ethos^®^ system (Varian, Palo Alto, CA, USA) were included in this study. Patients were treated with combined chemoradiotherapy and were registered on a prospective institutional clinical registry. The Ethics Committee of the University of Duisburg-Essen approved this study (18-8364-BO/23-11380-BO). The study was conducted according to the Declaration of Helsinki. All patients provided written informed consent. The oART treatment workflow begins with a cone beam CT (CBCT1). The system automatically contours pelvic organs at risk using an AI-based algorithm. These contours must be checked by the radiation oncologist, corrected if necessary and approved. Elastic deformable image registration (DIR) deforms the planning CT (PCT) onto the CBCT1, thereby generating the synthetic CT (sCT) of the day corresponding to the patient’s actual anatomy as observed on CBCT1. This sCT incorporates Hounsfield unit (HU) values derived from the PCT. By using a contour-based DIR, the deformed clinical target volume (CTV) of the day is then generated from the CTV in the PCT. Subsequently, the deformed CTV is presented to the radiation oncologist for correction and approval. Treatment plan optimization is then performed, and the user can choose between two treatment plans: the scheduled plan, which is calculated based on the actual anatomy, and the adaptive plan, which is optimized for the specific anatomy of the day. In the final steps prior to dose delivery, a second cone beam CT (CBCT2) is acquired for position verification. This enables a rigid registration with the reference CBCT1 and allows a corresponding adjustment of the treatment couch to partially compensate for patient movements or anatomical changes that may have occurred during the adaptation process.

### 2.2. Contouring

The contours of the rectum, bladder and CTV in CBCT1 were those approved during the clinical treatment sessions within the Ethos system. In the case of CBCT2, the contours for these structures were generated offline using an artificial intelligence-based auto-segmentation algorithm (MIM ProtégéAI^®^, version 1.1.3, MIM Software Inc., Cleveland, OH, USA). These segmented structures underwent a review and, where deemed necessary, were further modified by two experienced radiation oncologists. For delineating the CTV_CBCT1_, we employed two contour-based DIR methods within the MIM Maestro^®^ software (version 7.3.2, MIM Software Inc., Cleveland, OH, USA). DIR1 utilized the bladder and rectum as contours to steer the deformation, while DIR2 used only the bladder. In the first step, we employed DIR1 to transfer the CTV_CBCT1_ from CBCT1 to CBCT2. The resulting contours were reviewed, and any required corrections were made while comparing the CTV on both CBCT1 and CBCT2 side by side. Subsequently, in the second step, a third radiation oncologist examined the new CTV on CBCT2. This evaluation included reviewing the superposed CTV on CBCT2, which had been corrected by the first reviewer, as well as the CTV on CBCT2 deformed by both DIR1 and DIR2. Additionally, the CTV_CBCT1_ were presented side by side. Modifications suggested by reviewer 3 were discussed among all three reviewers and the final CTV_Final_ was consented by all three reviewers. The final contour-based DEF3 was generated using the CTV and the bladder contour on both CBCT1 and CBCT2.

### 2.3. Modelling of the Bladder and CTV Volume at Different Time Points

Contour-based deformation from CBCT1 at the start of the session (time point t_1_ = 0 min and V_1_ represents the bladder volume at t_1_) to CBCT2 (t_2_ = clinical adaptation time, V_2_ = the bladder volume at t_2_) was performed using DIR3 with bladder and CTV as steering contours, respectively. The deformation vector field was scaled for the generation of the bladder and the CTV at another time point t_i_. The scaling was accessed by the same time-dependent factor at all surface points assuming linear bladder volume increase over time as a first-order approximation. The relative length of a deformation vector at t_i_ > t_1_ in comparison to the time t_2_ is expressed as follows:

$$K=\frac{\sqrt[{3}]{1+\frac{\left(t_{i}-t_{1}\right)*\left(V_{2}-V_{1}\right)}{\left(t_{2}-t_{1}\right)*V_{1}}}-1}{\sqrt[{3}]{\frac{V_{2}}{V_{1}}}-1}$$

The scaling as well as the generation of the new deformation vector fields were assessed in MATLAB (R2022a). Figure S1a illustrates the bladder and CTV structures at a time point of 10 min between the fix timepoints t_1_ = 0 min and t_2_ = 18.8 min, achieved by adjusting the deformation vector field using the factor K at t_i_ = 10 min.

### 2.4. Equivalent Uniform Dose

The equivalent uniform dose (EUD) for an inhomogeneous dose distribution delivered to a tumor or an organ is the homogeneous dose resulting in the biological effect [27]. In this study, the generalized equivalent uniform dose (gEUD) based on a power law model was used [28]. For tumors, we used the parameter value a = −20, which is adequate for rather aggressive tumors and gives larger weight cold spots [29]. Furthermore, the gEUD values were normalized to the prescribed dose, and these values were referred to as nEUD.

### 2.5. Dose Accumulation

For each fraction, we performed a contour-based deformable registration between the CTV delineated on the PCT as the primary reference and the CTV_t1_ as well as CTV_t2_ observed on CBCT1 at t = 0 min and on CBCT2 after clinical adaptation time, respectively. An in-house script developed within the MIM^®^ environment facilitated this registration process. The script initiated with a rigid fusion between CBCT1 or CBCT2 and the planning CT, utilizing the isocenter positions from the scheduled plans as the initial alignment. Dose distributions for each fraction were subsequently deformed and accumulated, maintaining a voxel resolution of 1 mm per voxel in all spatial directions.

### 2.6. Dependence of the EUD on Margin around CTV

The CTV contours used in this study were derived from the clinically used PTV contours. To investigate the dependence of EUD on the CTV margin, the PTV was first contracted by applying an isotropic margin, ranging from 2 mm to 13 mm in 1 mm increments. Subsequently, EUD values were calculated for each resulting CTV. In the clinic, individualized non-isotropic margins were employed ranging from 5 to 10 mm and the results for the clinical CTV were described in a previous report [26]. All analyses in this study were performed with CTV volumes generated with 5 mm margins, unless otherwise specified. Specifically, CTV volumes generated with 5 mm margins were utilized for the contour-based DIR process.

### 2.7. Statistics

Statistical analyses were performed using SAS statistical software (SAS/STAT version 15.1, SAS Institute Inc., Cary, NC, USA). The empirical distribution functions from the different treatment series were compared with a nonparametric Kruskal–Wallis test using the procedure “npar1way”. Logistic regression was performed using the procedure “logistic”. Analyses of adaptation time-dependent trends of the nEUD values were performed with a general linear model. Depending on the error structure, especially the homogeneity of variances across adaptation times, regression on ranks or mixed models was used. Regression on ranks was performed with SAS procedures “GLM”, ranking the nEUD values from the different dose fractions per patient. For more quantitative analyses in the case of inhomogeneous error variances over adaptation time, a mixed linear model using fixed and random effects was used. Then, repeated measures analyses at different time points per fraction were performed with a mixed, fixed and random effect linear model using the procedure “mixed”. It allows for both heterogeneous variances and correlations between error components. Deviations from a normal distribution were analyzed using the Shapiro–Wilk test (Procedure Univariate, SAS). All statistical tests were performed as two-sided tests.

## 3. Results

Nine focal bladder cancer radiotherapy series from nine consecutive patients were analyzed for the effect of adaptation time on the nEUD values for the CTV (nEUD_CTV_). In the PCT, the target volume had a median of 226.32 mL (range: 79.52–487.51 mL) and the bladder volume had a median of 249.75 mL (range: 113.23–381.58 mL). The median overall adaptation was 18.8 min (range: 8.6–33.3 min). For five series, we delivered an intensity-modulated radiation therapy (IMRT) plan, while for the remaining four series, we used volumetric modulated arc therapy (VMAT). There was a significant difference in treatment duration between these techniques (*p* < 0.001, Kruskal–Wallis Test). The median, minimum and maximum treatment times for IMRT were 15.85 min (ranging from 8.6 to 26.68 min), while for VMAT, these values were 20.76 min (ranging from 13.23 to 33.3 min).

**Figure 1:**
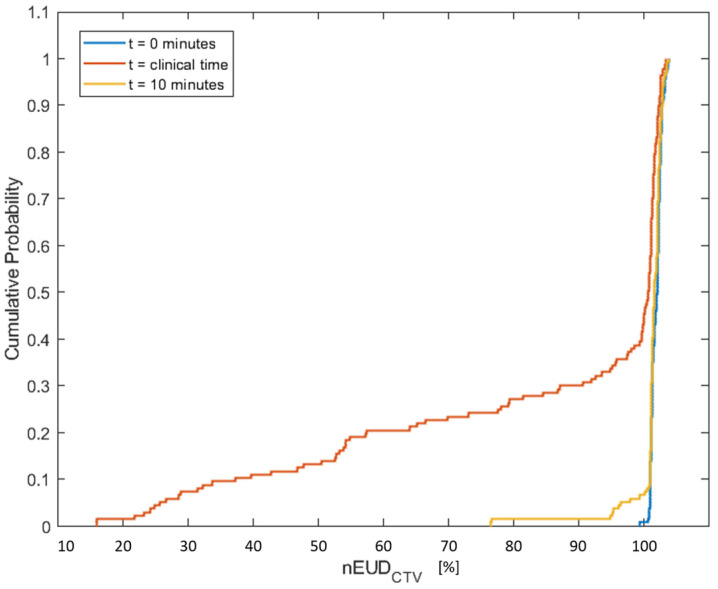
Empirical cumulative distribution of the equivalent uniform doses normalized to the prescribed dose for the CTV with a margin of 5 mm in the nine treatment series using the adaptive plan on CBCT1 at t_1_ = 0 min adaptation time (blue), on CBCT2 at the clinical adaptation time t_2_ (orange) and for the model-based CTV generated for an adaptation time of 10 min (yellow).

As a starting point, we analyzed the cumulative distribution functions of the nEUD_CTV_ values using the adapted plan to the anatomy in CBCT1 at the times t_1_, representing the time of the pre-treatment CBCT1, t_2_ of the post-adaptation CBCT2 and t_10_, i.e., 10 min after t_1_. The empirical distributions of the nEUD_CTV_ values for the 137 dose fractions using a uniform margin of 5 mm are shown in Figure 1. The nEUD values differed significantly between the times t_2_ and t_10_, as well as t_2_ and t_1_ (*p* < 0.001, Kruskal–Wallis Test). The proportion of treatment fractions falling below 95% of the prescribed dose over the three time groups was as follows: 0% at t_1_, 2.19% at t_10_ and 33.57% at t_2_.

**Figure 2:**
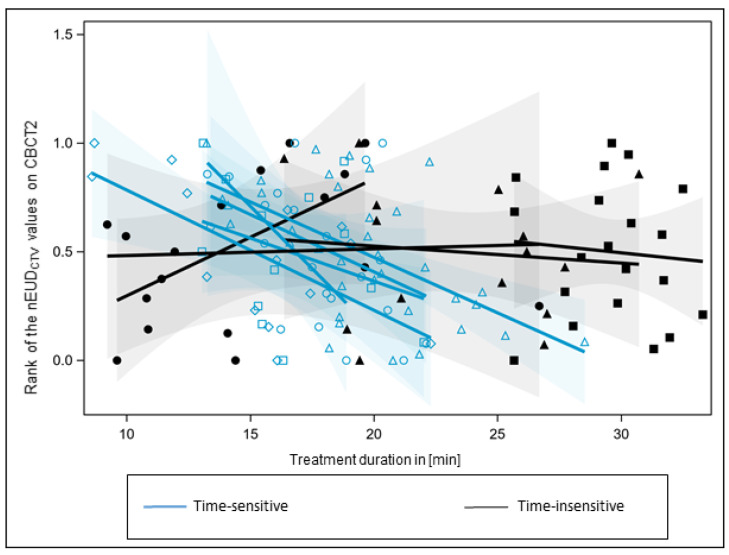
Trend analysis of the nEUD values for the CTV on adaptation time for the nine treatment series. nEUD_CTV_ values were ranked for each series and the rank numbers were scaled to the interval [0, 1]. There was a significant inter-series heterogeneity of slopes (*p* = 0.0157, F-test). According to the overall mean slope, series were ranked as time-sensitive (blue regression lines), with smaller slops than the overall slopes, or time-insensitive, with larger slops (black regression lines). Ranked nEUD_CTV_ values from a time-insensitive series were labeled by the same black filled symbol, and values from a time-sensitive series by the same blue open symbol. The 95% confidence limits of mean-predicted values are given as transparent confidence bands.

Next, the nine treatment series were analyzed with respect to an nEUD_CTV_ dependence on adaptation time (Figure 2). As residuals from a general linear model were not homogeneously distributed over adaptation time, values of the nEUD_CTV_ were ranked per patient and the rank numbers were scaled within the [0, 1] interval. An analysis of covariance was performed on the rank-transformed data, as the residuals were not normally distributed for the untransformed nEUD_CTV_ data (*p* < 0.0001, Shapiro–Wilk test), and the variance of the residuals was not independent of adaptation time. However, both assumptions were met when analyzing the transformed data. An overall effect between the dependence of nEUD_CTV_ values on adaptation time and the respective radiotherapy series was found to be significant (*p* = 0.0037, F-test). However, there were significant differences in time dependencies from series to series (*p* = 0.0157, F-test). The series were categorized into five time-sensitive series, with slopes smaller than the overall mean slope of −0.0262 ± 0.0080 min^−1^, and four time-insensitive series, with slopes larger than the overall mean slope (Figure 2). No heterogeneity in slopes remained between time-sensitive series (*p* = 0.84, F-test). The nEUD_CTV_ values on CBCT2 were smaller for the time-sensitive than for the insensitive series, indicating that the CTV was more deformed over time in time-sensitive compared to time-insensitive series (median nEUD_CTV_: 0.9969 vs. 1.0113; *p* = 0.0016, Kruskal–Wallis test). The same holds for the bladder volume on CBCT1, which was smaller in time-sensitive series (median bladder volume: 109.8 mL vs. 254.8 mL; *p* < 0.0001, Kruskal–Wallis test). In addition, we observed slightly larger dorsal deviations of the CTV in time-sensitive compared to insensitive series (median dorsal Hausdorff distance between the CTV in CBCT2 and CBCT1: 6.0 mm vs. 5.0 mm; *p* = 0.0363, Kruskal–Wallis test). The latter could, in part, be caused by a rectal balloon, which was used in three of the four time-insensitive but for none of the time-sensitive series. However, adaptation times were not larger but smaller in time-sensitive than in time-insensitive series (median adaptation times: 18.1 min for time-sensitive vs. 25.7 min for time-insensitive series; *p* < 0.0001, Kruskal–Wallis test).

For contour propagation of CTV or bladder, a time-dependent scaling factor for the deformation vectors from CBCT1 to CBCT2 was used. These volumes were generated at various time points per fraction: at 10 min after t_1_ and the medians of adaptation times within the lower, middle and upper terciles of all adaptation times, i.e., the 16.67th, 50.00th and 83.33th percentiles of all clinical adaptation times. The 16.67th percentile and its 95% confidence interval had a value of 14.14 min (95%CI: 13.24–15.57 min), the 50.00th percentile of 18.82 min (95%CI: 18.19–19.82 min) and the 83.33th percentile of 26.69 min (95%CI: 25.14–29.10 min). This time-dependent deformation model was highly fraction-specific, as can be shown by the intrafraction movement of selected points on the CTV surface by the respective deformation vector, e.g., the intrafraction posterior movement of the most posterior point on the CTV surface at an adaptation time of 18.82 min that is influenced by intrafraction bladder and rectum deformation (Figure S1b). The 50% confidence intervals for a prediction of this movement in a future fraction from a single past fraction are considerably larger than the median deviation over a treatment series. Therefore, the intrafraction deformation model cannot be predicted precisely from a single treatment session or a single planning CT session. The correlation of outward movements between points on different sides the CTV surface was only moderate, e.g., the Spearman correlation coefficient between the outward movements of the most superior and most posterior point of the CTV was rs = 0.35 [95% CI: 0.19–0.49].

**Figure 3:**
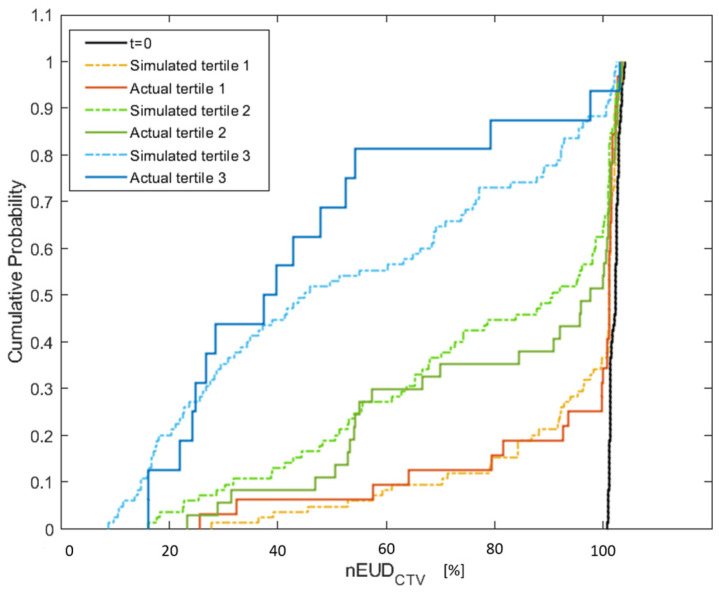
Empirical cumulative distribution of the nEUD_CTV_ values from the adaptive plans presenting a comparison between the actual nEUD values and the nEUD values calculated for the CTV at the simulated time points using the digital bladder model. The solid lines represent the observed nEUD_CTV_ values with a treatment duration belonging to the terciles T1 (red line), T2 (green line) and T3 (blue line). The dashed lines indicate the nEUD values for the model-based time-dependent CTV volumes at the median adaptation times in T1 (orange), T2 (green) and T3 (blue), i.e., 14.14 min, 18.82 min and 26.69 min. The distribution of the nEUD_CTV_ values on CBCT1 at t = 0 min is indicated for comparison (black).

In the subsequent analysis, we focused exclusively on the time-sensitive series of dose fractions. Figure 3 illustrates the empirical cumulative distribution functions of the nEUD_CTV_ values on CBCT2 for the five time-sensitive series according to the terciles in which the associated clinical adaptation time falls. There were significant differences between the nEUD_CTV_ values between the terciles (*p* = 0.0005, Kruskal–Wallis test). Pairwise comparisons revealed significant differences between all terciles: terciles 1 and 2, terciles 1 and 3, as well as terciles 2 and 3 (*p* < 0.0025, Kruskal–Wallis tests). In addition, the cumulative distribution functions of the nEUD_CTV_ values for the interpolated CTV volumes from each dose fraction of the time-sensitive series were depicted in Figure 3 at median adaptation times of the respective percentiles. The distributions of the nEUD values for the observed CTVs and the modeled CTVs did not differ within each percentile (*p* > 0.25, Mann–Whitney U-test).

**Figure 4:**
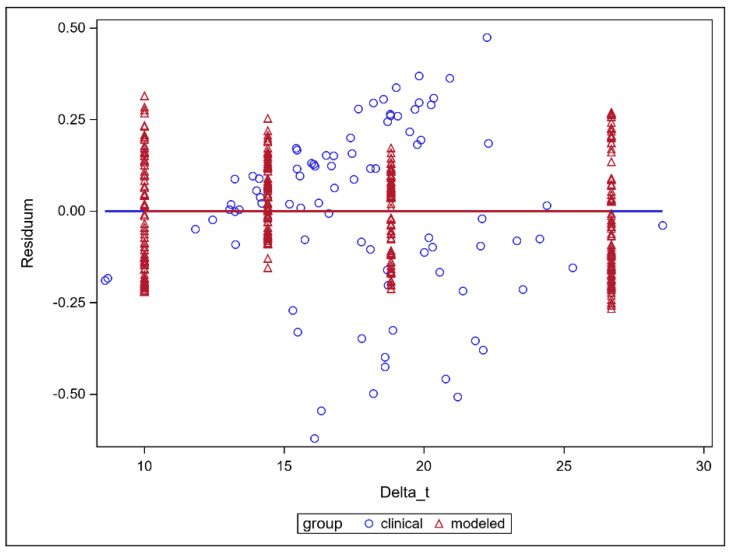
Residual nEUD_CTV_ values for the CTV volumes from a mixed linear model adapted to the nEUD values for the CTV volumes on CBCT2 after clinical adaptation times from fraction to fraction and to the interpolated CTV volumes at 4 distinct time points: 10 min, 14.14 min, 18.82 min and 26.69 min. The intrafractional dependence of the nEUD_CTV_ values on the adaptation time was modeled using the interpolated CTV volumes at the above 4 distinct time points. The nEUD_CTV_ values were explained by the respective time as a continuous covariate and the respective dose fraction as a mean effect using a repeated measures design. The interfractional dependence of the respective nEUD_CTV_ values used the clinically observed adaptation times and the respective patient as explanatory variables.

The inter- and intrafractional adaptation time dependence of the nEUD_CTV_ values are presented in Figure 4 for the five time-sensitive series. As the residual nEUD_CTV_ values from the model were not homogeneously distributed across adaptation times delta_t, we utilized a mixed linear model with the respective patient as a main effect for the interfractional analysis and the respective dose fraction as a main effect for the intrafractional analysis. There was a significant time dependence (*p* < 0.0001, F-test) with no significant difference in slopes obtained for the interfractional dependence of the nEUD_CTV_ values on adaptation time using CTV volumes on CBCT2 at t_2_ compared with the intrafractional dependence using the intrafractional interpolated CTV volumes at 10 min, 14.14 min, 18.82 min and 26.69 min as repeated measurements per fraction (*p* = 0.1072, F-test). The slopes for the interfractional and intrafractional dependencies of nEUD_CTV_ values on time were −0.04345 ± 0.0086 min^−1^ and −0.0294 ± 0.0014 min^−1^. In Figure 4, the marginal residuals of nEUD_CTV_ values from the model are depicted as the differences between observed and predicted nEUD_CTV_ values.

The residual nEUD_CTV_ values for the different fractions at t_2_ on CBCT2 from the above time-dependent model were further analyzed for a correlation with local deformations of the bladder not captured by a linear volume expansion. Here, we analyzed the local Hausdorff distances between the bladder wall on CBCT1 and the bladder wall on CBCT2, overlapping with the CTV on CBCT1. These Hausdorff distances were adjusted by the subtraction of the average increase in the bladder, estimated by the difference of the radii of spheres that have volumes equivalent to the bladder in CBCT1 and CBCT2. Figure S2a shows the increase in bladder volume from CBCT1 to CBCT2 dependent on the time interval between both CBCT studies for the fractions from the five series. The mean slope was 4.29 ± 0.89 mL/min. The dependence of the residual nEUD_CTV_ values from the model on the adjusted local Hausdorff distances of the bladder wall is shown in Figure S2b. There was a significant dependence of the residual nEUD_CTV_ values on the adjusted local Hausdorff distances of the bladder wall leading to local distortions of CTV at t_2_ not described by the model (*p* < 0.0001, *t*-test). The Spearman correlation coefficient was −0.455 (95% CI: −0.607–0.265). However, Figure S2c shows that the intrafractional nEUD_CTV_ residuals were not dependent on the adjusted local Hausdorff distances (*p* = 0.34, *t*-test). The Spearman correlation coefficient was rs = 0.02 (95%CI: −0.089–0.123). This difference between inter- and intrafractional analysis is due to the fact that adjusted Hausdorff distance is determined per fraction and the intrafractional analysis adjusts to peculiarities of each fraction.

**Figure 5:**
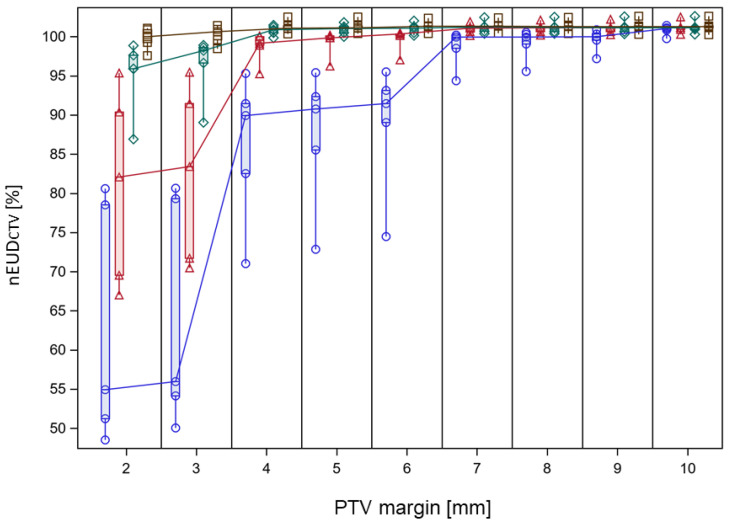
nEUD values for the CTV from the accumulated dose distribution of the adaptive plans on CBCT2 over a treatment series in dependence on PTV margin and adaptation time. Data are shown for the 5 time-sensitive treatment series. Open brown squares represent data modeled at 10 min adaptation time, green rhombus, red triangles and blue circles data at 14.1429 min, 18.877 min and 26.5866 min adaptation time. PTV margins were varied in 1 mm increments and data for the different adaptation times at the same integer PTV margin were separated by changing the horizontal position. This was carried out by varying the horizontal position around the respective PTV margin from the longest to the shortest adaptation time in submillimeter steps. Vertical box plots represent the indicated PTV margin and adaptation time extending from the 25th to the 75th percentile of values; the horizontal line inside the box represents the median of values and the whiskers indicate the whole range of values. Median nEUD_CTV_ values for the series at neighboring PTV margins were connected by drawn lines for each adaptation time.

**Figure 6:**
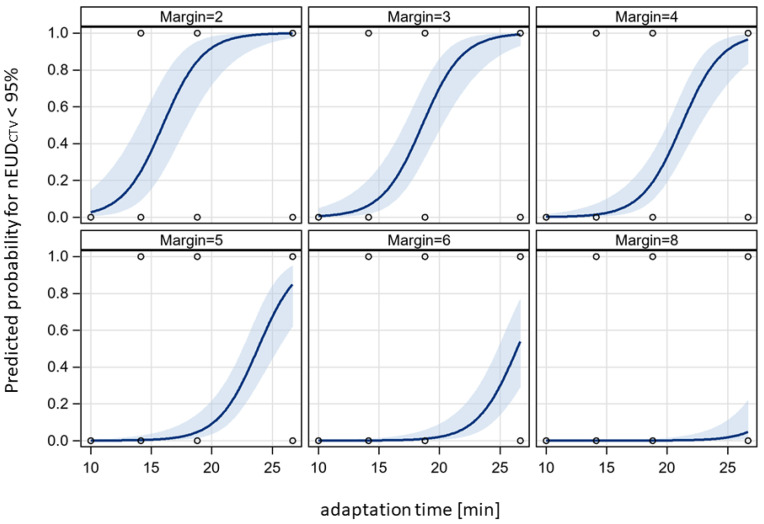
Adaptation time dependence of the probability of an nEUD_CTV_ < 95% for the accumulated dose distribution of a treatment series at different PTV margins. Data from Figure 5 were analyzed with a bivariate logistic model at an nEUD_CTV_ cut-off value of 95%. Logistic curves were given together with their 95% confidence bands. There was a significant effect of adaptation time and PTV margin on nEUD_CTV_ (*p* < 0.0001, Chi2 test for each variable).

In a next step, we analyzed the interplay between adaptation time and required PTV margin to maintain a nEUD_CTV_ value > 95% for the accumulated dose distribution over a treatment phase of adaptive dose fractions. The dose distributions adapted to the PTV in the initial CBCT1 for each dose fraction of the time-dependent treatment series were accumulated onto the planning CT using DIR-based dose accumulation, deforming the CTV volumes in CBCT2 at different adaptation times onto the CTV in the planning CT. Subsequently, the nEUD_CTV_ values were computed for the CTV volumes at the four time points: the adaptation times of 10 min, 14.1429 min, 18.877 min and 26.5866 min. In addition to the CTV with a 5 mm margin to the clinical PTV at t_1_ and t_2_, CTV volumes with margins ranging from 2 to 13 mm were generated through isotropic shrinkage of the PTV. The CTV volumes for different margin sizes were also propagated to the mentioned additional time points. The nEUD_CTV_ values at the four time points in dependence on PTV margin are shown in Figure 5 for the accumulated dose distributions of the different treatment series. An increase in the nEUD_CTV_ values was observed with both prolonged adaptation time and increased margin. At the clinical times t_2_ per dose fraction and the 5 mm PTV margin, the nEUD_CTV_ for the accumulated dose distribution stayed above 95.0% and even 99.0% of the prescribed dose for eight of the nine series. For the remaining series, which were time-sensitive, it stayed at 91.5% when accumulating doses from all fractions and exceeded 99.0% when accumulating the doses from fractions within adaptation times falling within the lower two terciles of values, i.e., times shorter than 20.8 min. A more quantitative analysis was performed by logistic regression at an nEUD_CTV_ cut point of 95% in dependence of adaptation time and PTV margin as continuous covariates. Figure 6 shows the time-dependent predicted probability of an nEUD_CTV_ value falling below the cut point for various PTV margins within a treatment series. The probability increased with time and fell with margin (*p* < 0.0001, Chi2 test for both covariates). The regression coefficients were 0.6028 ± 0.1270 min^−1^ for time and −1.5777 ± 0.3474 mm^−1^ for PTV margin, with a covariance of −0.0366 min^−1^ mm^−1^. The intercept was −6.4676 ± 1.7066. From this, it follows that for every 5 min extension of adaptation time, an additional PTV margin of 1.9 ± 0.24 mm is required for compensation. The upper 95% confidence limits for the predicted probability of nEUD_CTV_ for a treatment series < 95% stayed below 5% up to adaptation times of 10.0 min, 13.0 min, 15.7 min, 18.3 min, 23.0 min and 27.2 min at PTV margins of 3 mm, 4 mm, 5 mm, 6 mm, 8 mm and 10 mm, respectively.

## 4. Discussion

Adaptation time can profoundly decrease the effectiveness of focal oART for bladder tumors treated with a filled bladder in order to spare the contralateral walls in about half of the treatment series as a result of this study. Quite a few studies analyzed the effect of adaptation time on the coverage of the CTV by a dose distribution adapted to the imaging at the beginning of each fraction for tumors located near the bladder. Berger et al. found in their simulation study on adaptive proton therapy for cervical cancer that dose coverage of the CTV decreased with adaptation time from 5 min to 15 min using a PTV margin of 5 mm, and that dose coverage was even more time-sensitive using a margin of 2.5 mm [30]. About 25% of the dose fractions had a minimum dose to the hottest 98% of the CTV (D98) below 91% and 82% of the prescribed dose at 5 min and 10 min adaptation time. This simulation study used the pre-treatment CBCT imaging from conventional IGRT per fraction and a bladder expansion model, derived from a population of previously treated cervical cancer patients, together with a motion model to simulate bladder-induced CTV deviations. However, no further intrafractional imaging to derive fraction-specific deformations was performed. Brennan et al., 2023, found a decrease in the intraprostatic gross tumor volume (GTV) dose coverage measured by the D98 with increasing bladder filling during adaptation time using integrated boost radiotherapy with 2 mm PTV margins on an MR-Linac [31]. In addition, some studies showed that dose coverage of the CTV in prostate cancer can decrease importantly in terms of D95-D98 during treatment times of about 30 min from initial imaging to the end of dose delivery on an MR-Linac at 3 mm PTV margins [32,33].

Additional studies have shown an impact of time on the movement of tumors or structures near the bladder during the patient on-couch time. The maximum bladder wall movements from the start of a treatment session increased intrafractionally with time, as found in the study by Nishioka using implanted fiducial markers and stereoscopic fluoroscopy [34]. Eijkelenkamp et al. analyzed the deformations of the GTV in dependence on adaptation time from repeated imaging on an MR-Linac during five dose fraction [35]. The margin around the GTV on the initial pre-adaptation MRI scan was isotropically expanded to encompass the GTV in the consecutive scans per fraction. Margins required to encompass 90% of the intrafractional GTV deformations at the indicated later times increased from 4.0 mm at 10–15 min to 6.4 mm at 30–35 min.

In this study, we applied an approach using contour-based elastic deformation for estimation of bladder or CTV shape and deformation at different time points assuming linear volume increase in the bladder with time. Using this model, we found the intrafractional dependence of the nEUD_CTV_ values on adaptation time comparable with the interfractional dependence for the time-sensitive treatment series. In addition, adaptation times from all series needed in the clinic were classified into three terciles, and no significant differences were found between the empirical cumulative distribution functions of the interfractionally observed respective nEUD_CTV_ values and the distribution of the nEUD_CTV_ values for the model-based CTV volumes per tercile. While the interfractional time dependence compares the time dependence of different set-up deformations at different days from fraction to fraction per patient, the intrafractional time dependence captures the specific deformation of the day between CBCT1 and CBCT2 and propagates it to the considered adaptation time point. Although the model has passed the comparison of the inter- and intrafractional time dependences of the nEUD_CTV_ values, it has to be pointed out that deformations of the bladder wall from a linear dependence on bladder volume over time have been observed. Contractions of the bladder wall, the pelvic floor and the transversus abdominis muscle can also cause shifts of the bladder neck [36,37]. Accordingly, monitoring of prostate position with the Calypso electromagnetic tracking system showed rapid displacements within seconds that could relax afterwards but standard deviations of displacement within 5 min stayed below 2 mm for most fractions [38,39].

This study showed that the effect of prolonged adaptation time beyond 10 min on the nEUD of the accumulated dose distribution can be outweighed by an increase in the PTV margin of 1.9 ± 0.24 mm per 5 min increase in the time-sensitive half of treatment series. These findings underline that adaptation times have clinical significance not only for focal radiation therapies of bladder cancer but also for target volumes associated with the bladder contours, as for adaptive external beam boost therapies for cervical cancers not suited for brachytherapy or hypofractionated radiation therapies of prostate cancer [7,40]. For such dose-escalated or hypofractionated therapies, PTV margins are determinants of treatment-associated toxicities and the results of the MIRAGE trial for prostate cancer patients showed that an increase in a PTV margin of 2 mm results in a measurable increase in toxicity [40]. In this study, dose accumulation of all dose fractions per series revealed satisfactory results, with an nEUD > 95% in eight out of nine treatment series by a PTV margin of 5 mm. Mitchell et al. treated bladder cancer with adaptive whole bladder radiotherapy, with patients having emptied bladders before treatment, on an MR-Linac. They used PTV margins of 15 mm anteriorly and superiorly, as well as 5–10 mm in the other directions [1]. They found that with an increased workflow duration up to 60 min, there was an increase in the percentage of dose fractions for which a rigid adaptation of the table position as post-adaptation was required according to the verification imaging to recenter the target with the PTV [24]. In addition, during two percent of the dose fractions, patients had to leave the treatment room following adaptation and before treatment in order to void bladder or rectum at adaptation times above 45 min. However, an empty bladder does not allow for an optimal bladder wall sparing using focal bladder tumor radiotherapy due to the smaller distance of the uninvolved wall from the target [41]. In this study, we found that time-insensitive treatment series had larger bladder volumes and higher nEUD_CTV_ values than time-sensitive series. A similar finding was reported by Berger et al. for cervical cancers for which larger bladder volumes of the day are associated with smaller degradation of the effective dose to the CTV in terms of the D98 [30]. Therefore, attempts should be taken at implementing protocols for full bladder with a volume of about 250 mL, which in addition would allow better bladder wall sparing.

Limitations of this study include the smaller number of patients and that the anatomy was not monitored intrafractionally at multiple time-points, since this would cause extra dose exposure to the patient using CBCT imaging. In addition, extra 3D imaging takes extra time; MRI takes about 2 min for T2-weighted images [24]. From this study, it follows that adaptation times in the upper tercile of observed values above 20.8 min are critical and should be minimized. One option to speed up the process is the use of 9 or 12 field IMRT instead of VMAT plans, with optimization times of about 3–4 min instead of 13–14 min [42]. In addition, each additional imaging requires contouring of the organs at risk and of the target volume. In this study, target volume contouring on the verification CBCT was performed in a two-stage process by three physicians. In the future, efforts should be aimed at increasing automatization of on-line contouring using convolutional neuronal networks [43,44] which also might be patient-specific and trained on imaging studies from previous dose fractions of the considered patients [45].

## 5. Conclusions

Adaptation time should be aimed at being maintained below 20.8 min for pelvic tumors near the bladder to minimize margins around the CTV. There was a close dependence of PTV margin on adaptation time beyond 10 min of 1.9 ± 0.4 mm per 5 min adaptation time, in order to maintain the nEUD_CTV_ for the accumulated dose distribution per treatment series. A full bladder of about 250 mL can stabilize the anatomical scenario.

## Data Availability

The datasets generated and analyzed during the current study are not publicly available due to the right of personal data protection but can be made available by the corresponding authors upon reasonable request.

## Supplementary Materials

The following supporting information can be downloaded at: https://www.mdpi.com/article/10.3390/cancers15235629/s1, Figure S1a: Time-dependent model derived from the deformation vectors between CBCT1 and CBCT2 illustrating temporal changes in bladder and CTV volumes; Figure S1b: Intrafraction posterior (+)–anterior (-) movement of the most posterior point of the CTV in the planning CT within 18.8 min, the median adaptation time, from the time dependent deformation model; Figure S2a: Increase of the bladder volume in CBCT2 compared to CBCT1 with adaptation time for the dose fractions of the time-sensitive series; Figure S2b: Dependence of the residual nEUD_CTV_ values from the model shown in Figure 4 for the CTV volumes on CBCT2 underlaying the inter-fractional analysis; Figure S2c: Dependence of the residual nEUD_CTV_ values from the model shown in Figure 4 for the inter-fractional analysis of model-based CTV at adaptation times of 10 min, 14.14 min, 18.82 min, and 26.69 min.
