# Supplementary material for: Adaptation Time as a Determinant of the Dosimetric Effectiveness of Online Adaptive Radiotherapy for Bladder Cancer
Source: Cancers (Basel). 2023 Nov 29;15(23):5629. doi: 10.3390/cancers15235629 (PMC10705074; doi:10.3390/cancers15235629)
Supplement: Supplementary file 1 [file cancers-15-05629-s001.zip › cancers-2719197-supplementary.pdf]

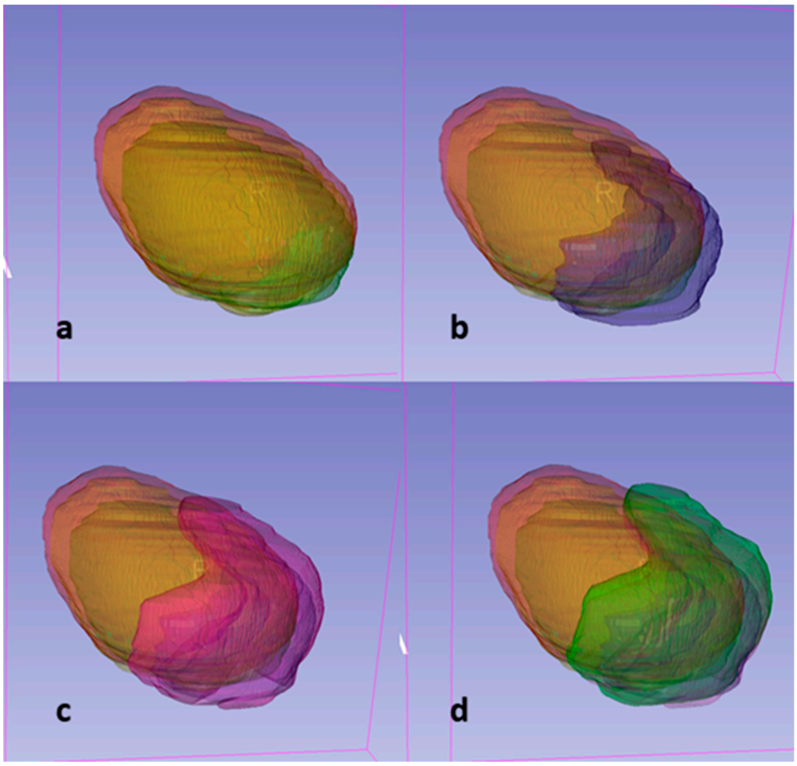


Figure S1a: Time-dependent model derived from the deformation vectors between CBCT1 and CBCT2 illustrating temporal changes in bladder and CTV volumes. In Panel (a), the bladder is depicted in green on CBCT1 at t1=0 minutes, in orange at the modelled time t=10 minutes, and in pink at the clinical time, here 18.8 minutes. The growth of the CTV at time points 0 minutes, 10 minutes, and 18.8 minutes is presented in Panels (b), (c), and (d), respectively.

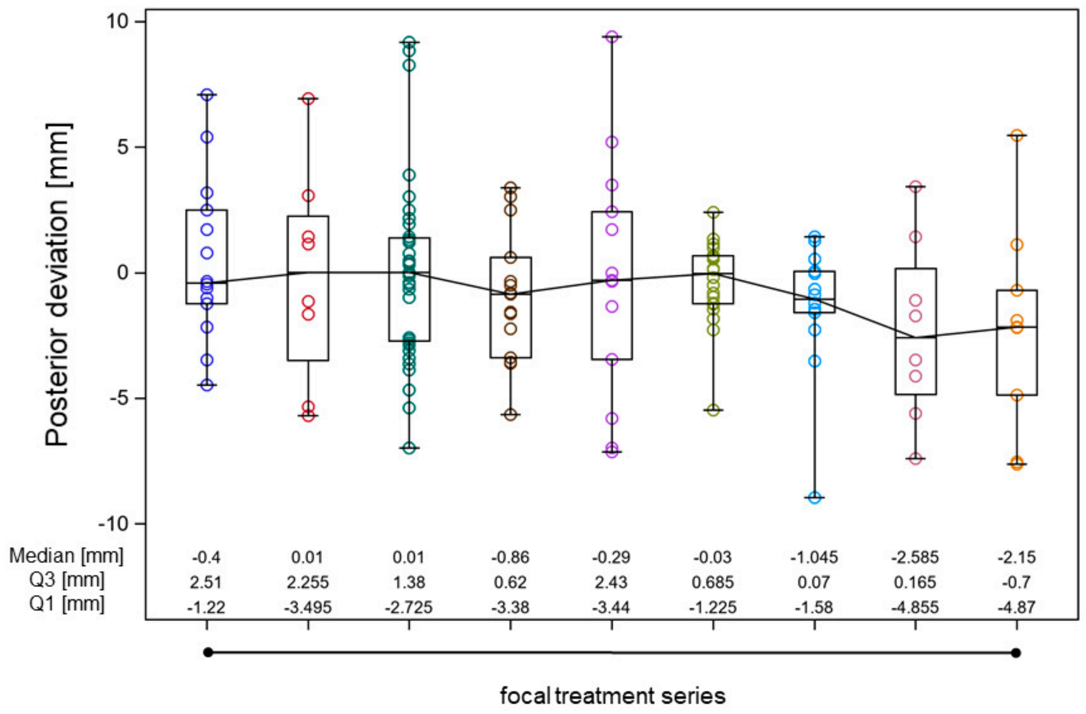


Figure S1b: Intrafraction posterior (+) – anterior (-) movement of the most posterior point of the CTV in the planning CT within 18.8 minutes, the median adaptation time, from the time dependent deformation model. The most posterior point of the CTV in the planning-CT was projected to all CTV volumes on CBCT1 by the inverse of the elastic deformation used for dose accumulation and then moved by the time dependent deformation model. Box plots are given for the posterior-anterior movements of the most posterior point of the CTV from the different fractions of each treatment series. The whisker lengths indicate the 95% confidence intervals. In addition, the median and the 50% interquartile range of the posterior movements are displayed at the bottom of the figure for each treatment series. Q1, Q3: lower and upper quartile of the distribution of posterior deformations for a treatment series. Medians posterior deformations of the treatment series were connected by lines.

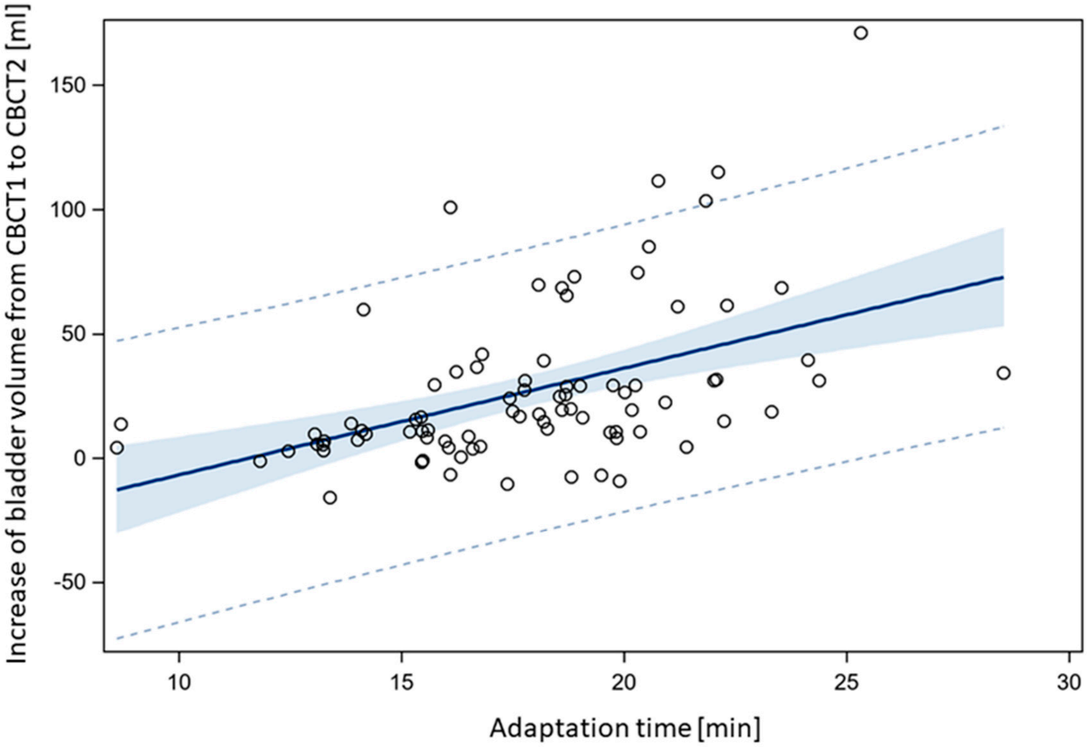


Figure S2a: Increase of the bladder volume in CBCT2 compared to CBCT1 with adaptation time for the dose fractions of the time-sensitive series. There was a significant time dependence ($p<0.0001$, t-test). The mean slope was 4.29 ± 0.89 ml /min.

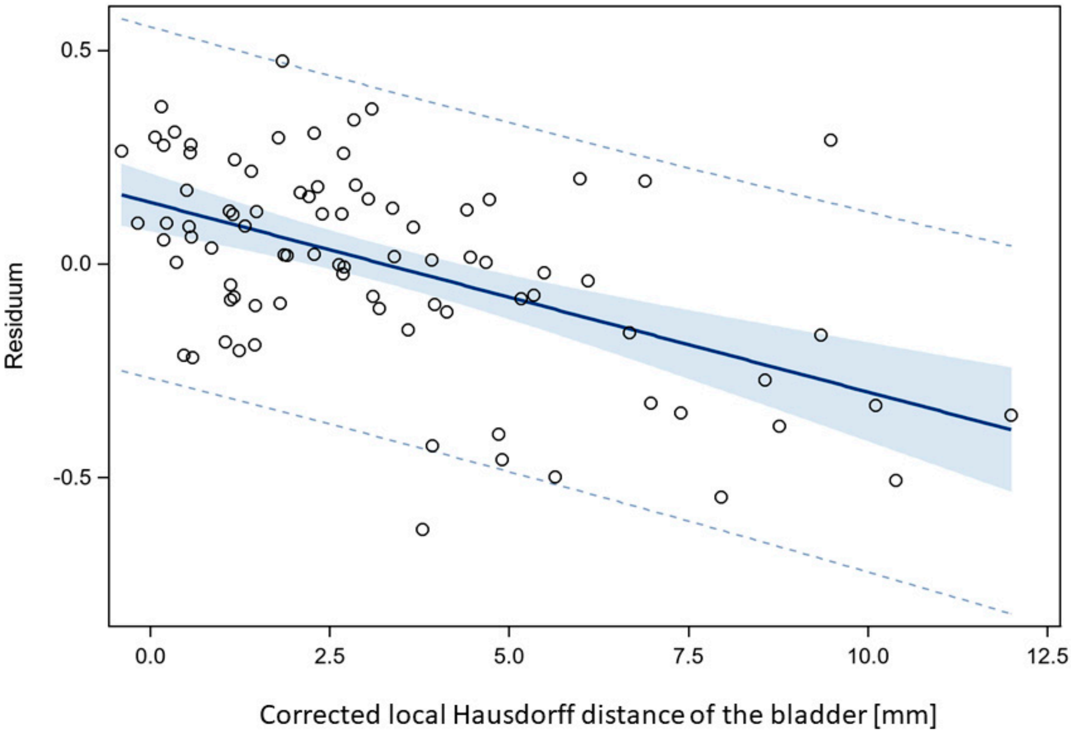


Figure S2b: Dependence of the residual $nEUD_{CTV}$ values from the model shown in Figure 4 for the CTV volumes on CBCT2 underlaying the inter-fractional analysis. The residuals were significantly dependent on the local Hausdorff-distances between the bladder wall within the CTV on CBCT1 and the bladder wall on CBCT2. These local Hausdorff distances were adjusted by the subtraction of the average increase of the bladder, estimated by difference of the radii of spheres that have volumes equivalent to the bladder in CBCT1 and CBCT2. There was a significant dependence of the residual $nEUD_{CTV}$ values on the adjusted local Hausdorff distances ($p<0.0001$, t-test).

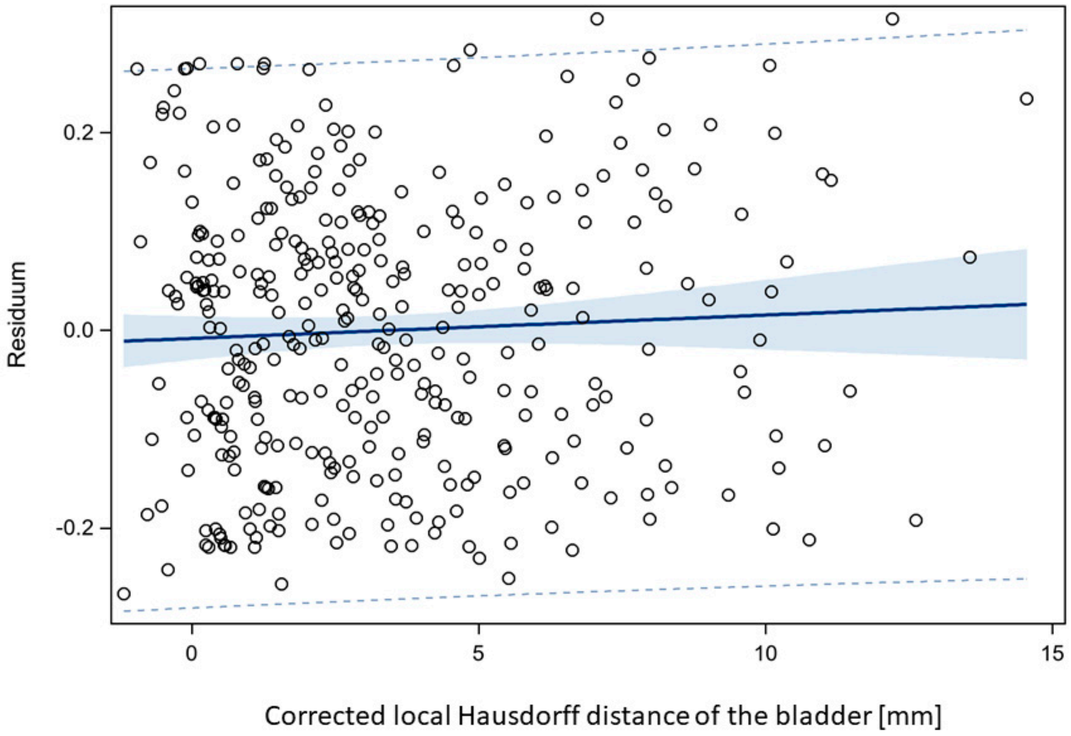


Figure S2c: Dependence of the residual $nEUD_{CTV}$ values from the model shown in Figure 4 for the inter-fractional analysis of model-based CTV at adaptation times of 10 min, 14.14 min, 18.82 min, and 26.69 min. There was no significant dependence of the residual $nEUD_{CTV}$ values on the adjusted local Hausdorff distances (p=0.34, t-test), the Spearman correlation coefficient was rs=0.02 (95%CI: -0.089 – 0.123).
